# Perspectives on the Impact of the COVID-19 Pandemic on Research: A Cross-Sectional Survey of Pediatric Otolaryngologists

**DOI:** 10.7759/cureus.60436

**Published:** 2024-05-16

**Authors:** Peter Kfoury, Jordan C Stout, Victoria J Palacios, Wei Yang, Nicole L Molin, Matthew A Firpo, Albert H Park

**Affiliations:** 1 Department of Otolaryngology - Head and Neck Surgery, University of Utah School of Medicine, Salt Lake City, USA; 2 School of Medicine, University of Nevada, Reno, USA; 3 School of Public Health, University of Nevada, Reno, USA; 4 Department of Sleep Medicine, Thomas Jefferson University Hospital, Philadelphia, USA; 5 Department of Surgery, University of Utah School of Medicine, Salt Lake City, USA; 6 Department of Otolaryngology - Head and Neck Surgery/Department of Pediatrics, University of Utah School of Medicine, Salt Lake City, USA

**Keywords:** pandemic, covid-19, otolaryngology, pediatric otolaryngology, research

## Abstract

Objective: To investigate the perspectives of pediatric otolaryngologists on the impact of the coronavirus disease 2019 (COVID-19) pandemic on their research.

Methods: Two surveys were sent to members of the American Society of Pediatric Otolaryngology (ASPO) in 2019 and 2021 to assess research perspectives before and during the COVID-19 pandemic. The surveys contained questions about research engagement, barriers, time allocation, and shifts in research focus.

Results: The COVID-19 pandemic reshaped research within pediatric otolaryngology, with a mixed impact on the amount of time allocated to research endeavors. Almost half of respondents reported a change in research focus to COVID-19-related studies. Protected time and funding were significant pre-pandemic barriers, whereas reduced staff, collaboration opportunities, and enrollment limitations emerged as key pandemic-related obstacles. A personal commitment to research was most strongly correlated with time spent on this endeavor. During the pandemic, women were less likely to report an increase in research activity when compared to men, possibly due to a disproportionate burden of caregiving on women during school closures and stay-at-home orders.

Conclusion: Overall, the pandemic prompted both increases and decreases in research time allocation, depending on individual circumstances and priorities. Despite new challenges, pediatric otolaryngologists remain committed to research and have continued to remain productive.

## Introduction

Medical advancements and research endeavors can go hand in hand to improve patient care. Physicians credit several advantages to research [1]: understanding the mechanism of a disease, testing new therapies, and enhancing standing in society. While the importance and benefits of research are known, barriers may dissuade physicians from engaging in the research process [2]. A survey of senior trauma surgeons in 2000 revealed that 38% of those involved in basic science research ended their research career by the age of 39 years [3]. Residents of surgical sub-specialties report a lack of time, statistical knowledge, research interest, and access to supervisors and mentors, as well as a lengthy research ethics approval process, as the greatest barriers in their research endeavors [4,5]. In a survey of 34 American Society of Pediatric Otolaryngology (ASPO) members, published in 2015, lack of time was reported as the most significant barrier to successful publication [6].

The COVID-19 pandemic changed the landscape of research through a new set of obstacles, including lockdowns and safety measures. From withholding non-COVID-19-related clinical trials to job losses and budget cuts, US academia came to a standstill [7,8]. Few studies have investigated how COVID-19 impacted research activities within otolaryngology. Some commented on the impact of the pandemic on the discontinuation of several clinical trials [9]. Others observed a highly positive impact on research output in otolaryngology, with publication rates increasing by 34.4% [10,11]. The purpose of this study is to investigate the perspectives of pediatric otolaryngologists on the impact of the COVID-19 pandemic on their research. We hypothesized that the COVID-19 pandemic reduced research engagement and time allocation for pediatric otolaryngologists, with a disproportionately larger effect on caregivers. This article was previously presented as a poster abstract at the American Academy of Otolaryngology-Head and Neck Surgery Forum (AAO-HNSF) annual meeting held in Philadelphia on September 10-14, 2022.

## Materials and methods

Study design

A survey (Appendix A) consisting of 22 questions related to research perspectives was sent to the American Society of Pediatric Otolaryngology (ASPO) members from August to December 2019. After the start of the pandemic, a second survey (Appendix B) of 22 questions with nine COVID-19-specific items was sent to ASPO members from September to December 2021 to investigate potential changing perspectives. The surveys contained questions about research engagement, barriers, time allocation, and shifts in research focus. Both surveys were reviewed and accepted for submission by the ASPO committee prior to distribution. They were emailed to all ASPO members. The results were collected on a Google Forms document for analysis. Surveys with more than three incomplete questions or when participants opted out of the questionnaire were excluded. Institutional Review Board (IRB) exemption was obtained from the University of Utah School of Medicine (Approval number: 00110018).

Statistical analysis

Intragroup and intergroup differences in responses were analyzed using Fisher’s exact test, Chi-square test, odds ratios (ORs), multiple logistic regressions, and descriptive statistics. Statistical significance was defined as a p-value less than 0.05. Statistical analysis was performed using Microsoft Excel.

## Results

Characteristics of the respondents

The demographic and research characteristics of respondents across both surveys are summarized in Table 1. The 2019 (pre-COVID-19) survey received 134 responses (18% response rate). One survey was abandoned halfway through, lacked a significant amount of information, and was excluded accordingly. A total of 133 surveys were included for data analysis. All respondents were pediatric otolaryngologists; one was retired. 

**Table 1:** Respondent demographics and research characteristics, n (%) ^¶^Fisher’s exact test was applied; *p<0.05; n: Sample Size

		2019 (n=133)	2021 (n=64)	p-value^¶^
Gender	Male	92 (69%)	41 (64%)	0.260
	Female	37 (28%)	23 (36%)	
	Prefer not to say	4 (3%)	0 (0%)	
Practice setting	Academic	118 (89%)	62 (97%)	0.062
	Non- Academic	15 (11%)	2 (3%)	
Years in practice	Less than or equal to 10 years	57 (43%)	29 (45%)	0.878
	Greater than 10 years	75 (56%)	35 (55%)	
	Blank	1 (1%)	0 (0%)	
Personal importance of research on a 5-point scale (5 is the highest)	Three or below	61 (46%)	26 (41%)	0.541
	Four or above	71 (53%)	37 (58%)	
	Blank	1 (1%)	1 (1%)	
Amount of time spent on research	No time for research	9 (7%)	1 (2%)	0.0013*
	Less than or equal to 10%	91 (68%)	31 (48%)	
	Greater than 10%	33 (25%)	32 (50%)	
Wish they had more time for research	Yes	79 (59%)	22 (34%)	0.0013*
	No	54 (41%)	42 (66%)	

The 2021 (mid-COVID-19) survey attained 72 responses from pediatric otolaryngologists (10% response rate). Eight participants opted out of completing the full survey. The remaining 64 surveys were included for data analysis. All respondents were practicing pediatric otolaryngologists, including one resident physician.

The majority of survey respondents practiced in an academic setting in both 2019 and 2021 (89% and 97%, respectively, Fisher’s exact test (FET), p=0.062). Participants in both surveys had comparable years of experience, with 56% of surgeons pre-COVID-19 and 55% mid-COVID-19 in practice for greater than 10 years (FET, p=0.878). Additionally, they had similar outlooks on the importance of research in their career, with 53% of respondents pre-COVID-19 and 58% mid-COVID-19 regarding their research as very important (4 or 5 on a scale of 1 to 5) (FET, p=0.541).

In contrast, there were significant differences in time spent on research and the desire for more research from 2019 to 2021. The proportion of respondents spending greater than 10% of their time on research approximately doubled from 25% pre-COVID-19 to 50% mid-COVID-19 (FET, p=0.001). A smaller proportion of respondents wished they had more time for research during the pandemic, with 59% of respondents who wanted more time for research pre-COVID-19, compared to 34% mid-COVID-19 (FET, p=0.001).

Research barriers

**Figure 1:**
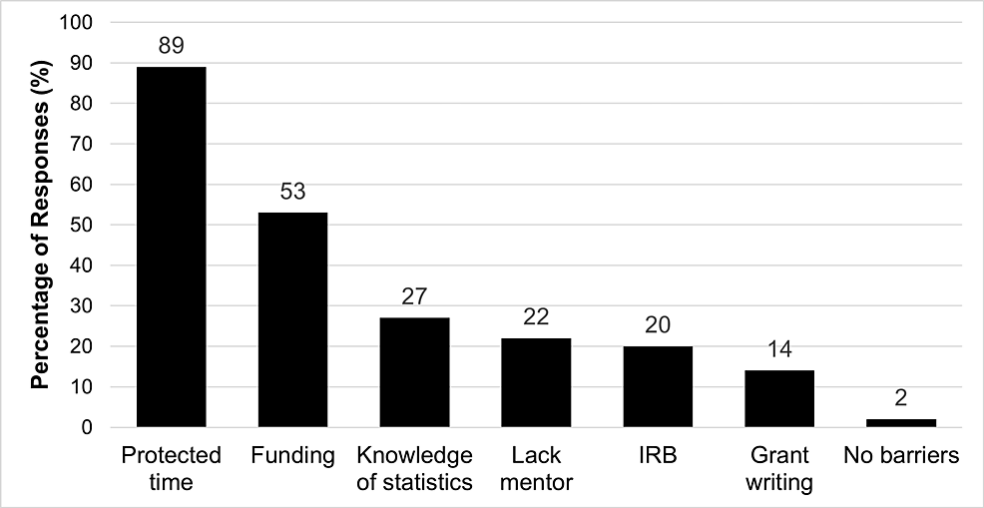
Perceived barriers to research for the 133 participants in the 2019 survey COVID-19: Coronavirus disease 2019; IRB: Institutional review board Participants were allowed to choose more than one answer.

**Figure 2:**
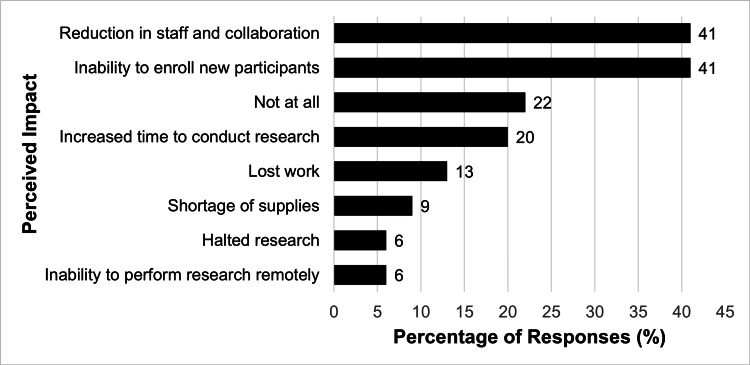
Perceived impact of COVID-19 on conducting research on the 64 participants of the 2021 survey COVID-19: Coronavirus disease 2019 Participants were allowed to choose more than one answer

Perceived barriers to research pre-COVID-19 are summarized in Figure 1. The most significant barriers to research cited by participants included protected time (89%) and lack of funding (53%). Only three respondents (2%) felt that there were no barriers to research. The impact of new obstacles to research during the COVID-19 pandemic, identified in the 2021 survey, is summarized in Figure 2. Reduction in staff and lack of opportunities for collaboration (41%), as well as the inability to enroll new participants (41%) were cited as the greatest barriers to research efforts during the pandemic. Interestingly, the third and fourth most frequent responses to this question stated that research efforts were “not at all” impacted (22%) or that the respondents had increased time to conduct research (20%).

Time spent on research

Table 2 summarizes the results from COVID-19-specific questions on the 2021 survey. A total of 39% of respondents reported a decrease in the amount of time allocated to research during the pandemic. However, 30% felt the pandemic did not alter the time spent on research, and another 30% even suggested that it increased their time allocated to research. Respondents were asked whether cancellation of conferences or lack of mentorship during COVID-19 affected their research efforts, with 48% responding “agree” or “strongly agree.” When asked whether fear of putting others and loved ones at an increased risk of transmission changed research aims, 20% responded “yes.” Additionally, 41% of respondents felt that their research focus had shifted to COVID-19-specific research, along with the continuation of non-COVID-19 research efforts.

**Table 2:** Questions in 2021 Survey evaluating the impact of COVID-19 on research efforts COVID-19: Coronavirus disease 2019

		Total (n=64)
Has the amount of time you spend on research changed since COVID-19?	Increase or Slight Increase	19 (30%)
	Unchanged	19 (30%)
	Decrease or Slight Decrease	25 (39%)
	Blank	1 (2%)
Has your research changed from non-COVID-19 to COVID-19 research?	Yes, with continued non-COVID-19 research efforts	26 (41%)
	Yes, with a complete halt of non-COVID-19 research	0 (0%)
	Not at all	38 (59%)
How has the added responsibility of being a caregiver during COVID-19 impacted your time dedicated to research?	More time spent on research (due to impact)	7 (11%)
	Less time spent on research (due to impact)	25 (39%)
	No impact	32 (50%)
Has fear of increased transmission of COVID-19 to others and loved ones changed your research aims?	Yes	13 (20%)
	No	51 (80%)
Has cancellation of conferences or lack of mentorship affected your ability to conduct research?	Agree or Strongly Agree	31 (48%)
	Neutral	19 (30%)
	Disagree or Strongly Disagree	12 (19%)
	Not applicable	2 (3%)

Factors associated with spending greater than 10% of time on research pre-COVID-19 are displayed in Table 3. Those who spent more than 10% of their time on research were more likely to be female (OR 2.8, p=0.02), felt that research was very important, defined as 4 or 5 out of 5 (OR 10.1, p<0.0001), had institutionally protected research hours (OR 3.8, p=0.004), and had funding (OR 4.8, p=0.0003). These variables remained significant when controlled for potential confounding variables using a multiple regression model.

**Table 3:** Factors that influenced spending greater than 10% of time on research in 2019 *p<0.05; CI: Confidence Interval

Variable		Odds Ratio (95% CI)	p-value
Gender	Female	2.8 (1.2, 6.5)	0.02*
	Male (Reference)		
Years in Practice	>10 years	1.1 (0.5, 2.4)	1.00
	0-10 years (Reference)		
Practice Type	Academics	1.4 (0.4, 5.3)	0.76
	Non academics (Reference)		
Research Importance on 5-point scale	>4	10.1 (3.3, 30.9)	<0.0001*
	<3 (Reference)		
Feels Basic Science Research has become harder	No	1.2 (0.5, 3.0)	0.81
	Yes (Reference)		
Feels Clinical Research has become harder	No	1.4 (0.6, 3.4)	0.49
	Yes (Reference)		
Has Funding	Yes	4.8 (2.0, 11.5)	0.0003*
	No (Reference)		
Salary related to having funding	Yes	1.2 (0.4, 3.6)	0.77
	No (Reference)		
Salary related to research productivity	No	1.3 (0.5, 3.1)	0.82
	Yes (Reference)		
Institution offers protected research time	Yes	3.8 (1.6, 9.1)	0.004*
	No (Reference)		

Factors associated with spending increased time on research mid-COVID-19 are displayed in Table 4. Participants who reported research as very important (OR= 16.3, p=0.021) or shifted research to COVID-19-specific topics (OR= 7.9, p=0.039) were more likely to spend an increased amount of time on research. Though not statistically significant, male researchers tended to dedicate more of their time to research compared to female participants (OR=5.0, p=0.085). Furthermore, when asked whether the responsibility of being a caregiver has impacted their time dedicated towards research, female participants were more likely to agree with this statement compared to men (one-tailed Chi-square test, p= 0.034).

**Table 4:** Factors that influenced having increased time for research during the COVID-19 pandemic * p<0.05; ^¶^ A majority of participants responded with “Not Applicable” on these three variables CI: Confidence Interval; N/A: Non-Available; n: Sample Size

Variable		Odds Ratio (95% CI)	p-value
Gender	Male	4.97 (0.80-30.86)	0.085
	Female (Reference)		
Years in Practice	0-10 years	2.13 (0.29-15.75)	0.457
	>10 years (Reference)		
Practice Type	Academics	8.43 (0.78-90.90)	0.079
	Non-academics (Reference)		
Percent of Time spent on research	<10%	2.87 (0.45-18.28)	0.264
	>10% (Reference)		
Research Importance on 5 point scale	>4	16.26 (1.52-173,91)	0.021*
	<3 (Reference)		
Feels Basic Science Research has become harder	Yes	n was too small to report^¶^	N/A
	No		
Feels Clinical Research has become harder	Yes	n was too small to report^¶^	N/A
	No		
Feels Funding has changed	Yes	n was too small to report^¶^	N/A
	No		
Feels the research focus has changed to COVID-19-specific research	Yes	7.89 (4.25-56.29)	0.039*
	No (Reference)		
Feels the responsibility of being a caregiver for loved ones due to COVID-19 impacted time dedicated toward research efforts	Yes	0.62 (0.27-3.71)	0.601
	No (Reference)		

Addressing barriers to research

**Figure 3:**
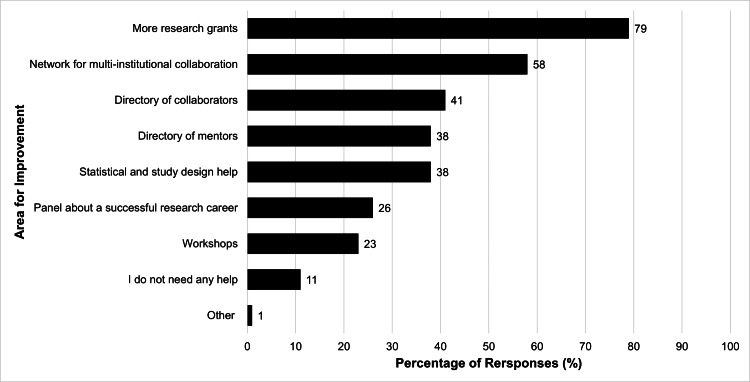
Responses when asked: "How can ASPO help your research career?” Participants were allowed to choose more than one answer

In the 2019 survey, respondents were asked how societies such as ASPO could help their research careers. Of the options listed, participants felt that ASPO could provide more grants for research (79%), create a network for multi-institutional collaboration (58%), establish a directory of potential collaborators (41%) and mentors (38%) with research interests and contact information, and provide statistical and study design assistance (39%). Fourteen respondents (11%) felt they did not need any help (Figure 3).

## Discussion

We hypothesized that the COVID-19 pandemic had reduced research engagement and time allocation for pediatric otolaryngologists, with a disproportionately larger effect on caregivers. Our results show that the picture is much more nuanced, with both increases and decreases in research allocation and a shift in focus to COVID-19-related research.

Despite a worldwide pandemic, a majority of pediatric otolaryngologists remained devoted to research. A personal commitment to research appears to be the greatest factor in the amount of time spent on this endeavor both before and during the pandemic. COVID-19 had a mixed impact on the amount of time allocated to research, though almost half of respondents shifted some of their efforts to COVID-19-related research. The barriers to research during COVID-19 were not surprising. They were linked to direct effects on staff reduction, as well as challenges with collaboration and enrollment of participants. During the pandemic, women were more likely to report that the responsibility of being a caregiver had impacted their time dedicated to research when compared to men. The cancellation of conferences and lack of mentorship were also important negative factors impacting research.

Before the pandemic, the lack of protected time was identified as the primary barrier to research among ASPO members (89%), which is consistent with prior studies, including a previous survey of ASPO members published in 2015 [6], as well as studies in other medical specialties [5,12]. The pandemic presented a new set of challenges. Reduced staff, decreased opportunities for collaboration, and a decrease in participant enrollment emerged as the predominant obstacles [7,9]. These new challenges are unsurprising given the rise of strict safety measures and lockdowns to curb the spread of the virus. Other studies within otolaryngology identified similar difficulties, finding that the pandemic prompted a decline in the publication of randomized clinical trials and meta-analyses [9,11]. The majority of discontinued trials reported recruitment problems, as well as a shift in resource allocation towards COVID-related research [9,11]. The disruptions encountered by pediatric otolaryngologists were also consistent with broader trends observed in other medical and research communities [13].

The allocation of research time during the pandemic revealed a mixed picture of the impact on pediatric otolaryngologists. While a significant proportion increased their research commitment, approximately 40% of survey respondents reduced their research time during the pandemic. The percentage of surgeons who allocated more than 10% of their time to research increased significantly, rising from 25% pre-COVID to 50% during COVID-19. The increased amount of time spent on research has translated to a higher number of publications, with a 34% increase in publication rates within otolaryngology [10,11]. This upsurge is mainly comprised of cohort and retrospective research that can be completed remotely and correlates with increases in both COVID and non-COVID-related articles [11]. These changes may be attributed to increased research productivity stemming from the adjournment of elective surgeries and the pandemic’s restrictions on clinical practice [14-16].

This study found that respondents were more likely to maintain or increase their research time during the pandemic. Additionally, those who reported an increase in time spent on research during the pandemic were statistically more likely to report a shift in research efforts to COVID-19-specific topics, along with continued non-COVID-19 research. This shift was likely driven by the urgency of addressing healthcare challenges related to COVID-19 [11,17].

Our survey indicated that female pediatric otolaryngologists were more likely to spend increased time on research than their male counterparts before the COVID-19 crisis. Other recent studies have shown that female otolaryngologists have been maintaining research productivity during their early career phases, possibly because of delayed childbearing and more equal sharing of household responsibilities with their partners [18-20]. Unfortunately, there may have been a backslide in the involvement of women in research during the pandemic. Though the odds ratio did not reach statistical significance, possibly due to the smaller sample size of the mid-COVID-19 survey, male respondents were more likely to report increased time spent on research since the start of COVID-19 than female respondents. This discrepancy may have been associated with a disproportionate burden of caregiving on women during school closures and stay-at-home orders. On the mid-COVID-19 survey, female participants were more likely to report that the responsibility of being a caregiver had impacted their time dedicated towards research when compared to men; a finding which did reach statistical significance. This pattern is consistent with observations in other papers, whereby women in academics, particularly those with children at home, were disproportionately affected by the pandemic [21]. The closure of schools and the enforcement of stay-at-home orders led to an increased burden of primary caregiving responsibilities on female researchers [22,23].

Nearly half of respondents reported that cancellations of conferences and lack of mentorship during COVID-19 affected their research efforts. The study’s findings emphasize the central role of personal commitment to research. Both before and during the pandemic, those who regarded research as very important in their careers were more likely to dedicate a substantial portion of their time to the endeavor.

These findings underscore that pediatric otolaryngologists are motivated and flexible in their research endeavors. Their insights on addressing barriers to research, captured in the pre-COVID survey, can offer helpful guidance for organizations to support these efforts. A significant majority of participants (79%) expressed a strong desire for increased grant opportunities, indicating a need for increased research funds. Their desire for multi-institutional collaboration reflects the value of partnerships in enhancing research quality and impact. The survey also revealed an interest in establishing directories for potential collaborators and mentors, as well as increased access to biostatistical training or resources. In 1999, the Association for Surgical Education established a funding initiative aimed at promoting innovative research to advance surgical education. This grant played a pivotal role in facilitating the publication of 70 articles by distributing a total of $988,000 between 1999 and 2013 [24]. Furthermore, many studies have found that multi-institutional collaboration positively correlates with increased quality and quantity of research outcomes [25,26]. Conversely, other studies underscore that a lack of adequate funding inhibits the production of quality research [27,28]. These findings emphasize the importance of fostering connections and providing the appropriate resources within the research community, facilitating knowledge exchange, and providing guidance to early-career researchers [29].

In light of the evident strain that the COVID-19 pandemic has placed on caregivers, particularly women, it is imperative to prioritize and allocate resources to facilitate their engagement in research activities. Recognizing the pivotal contributions that caregivers make to both family and society, it is essential that they have access to the necessary tools, support, and opportunities to pursue research endeavors. Funding organizations play a crucial role in advancing this cause. These organizations and societies could take a more proactive role by providing a comprehensive directory for collaboration, mentorship, and statistical support. By establishing a network that connects researchers, this directory could facilitate knowledge exchange, support systems, and mentorship opportunities.

Limitations

The lower response rate observed on the mid-COVID-19 survey, which yielded 64 complete responses, likely limited the ability to achieve statistical significance across multiple data points and potentially introduced a sampling bias. This could affect the generalizability of the findings and limit the extent to which our results accurately represent the broader population of pediatric otolaryngologists. Additionally, statistical comparisons between the 2019 and 2021 surveys were limited by changes in language and questions added to the 2021 survey to include COVID-19-specific topics. This was an unavoidable consequence of our inability to predict the COVID-19 pandemic in 2019.

## Conclusions

The COVID-19 pandemic has presented unique challenges and opportunities for research within pediatric otolaryngology. The challenges were linked to difficulties in patient recruitment, increased burden of caregiving responsibilities, decreased staffing, and decreased opportunities for collaboration. The pandemic had varying effects on researchers, with some reporting increases in research commitments and others reporting decreases due to the exceptional barriers encountered. Overall, pediatric otolaryngologists remain committed to research and have managed to remain productive despite these unforeseen circumstances.

## Appendices

Appendix A: Survey distributed in 2019 to ASPO members to investigate their perspectives on research

*  Required

1.     Email address *

a.      Your email

2.     How old are you? [years]

a.      Your answer

3.     When did you complete your training? e.g. 2005

a.      Your answer

4.     Gender

a.      Male

b.      Female

5.     Years in Practice

a.      0-5 years

b.      6-10 years

c.      11-15 years

d.      More than 15 years

6.     Please describe yourself

a.      Pediatric Otolaryngologist, Fellowship Trained

b.      Pediatric Otolaryngologist, Non-Fellowship Trained

c.      Pediatric Otolaryngology Fellow

7.     How would you describe your practice?

You may check more than one option

a.      Academic or University Private Practice

b.      Managed care

c.      Hospital setting

d.      Other:

8.     What percent of your time do you spend on research?

a.      Not at all

b.      1-10% of my time

c.      11-20% of my time

d.      21-50% of my time

e.      51-75% of my time

f.       More than 75% of my time

9.     What percent of your time would you like to spend on research?

a.      none

b.      1-10% of my time

c.      11-20% of my time

d.      21-50% of my time

e.      51-75% of my time

f.       More than 75% of my time

10.  How important is a research career to you personally?

Not Important

a.      1

b.      2

c.      3

d.      4

e.      5

Very Important

11.  The focus of my research is on (check all that apply)

a.      Retrospective review of components of clinical practice

b.      Prospective analysis of components of clinical practice

c.      Systematic review or meta-analysis of a clinical question

d.      Clinical trials

e.      Basic science

f.       Other:

12.    My field of research interest is in (check all that apply)

a.      Airway

b.      Hearing Loss

c.      Dysphagia

d.      Otology

e.      Professional Practice

f.       Outcomes

g.      Rhinology or Sinus

h.      Tonsil or Adenoids

i.       Head and Neck

j.       Sleep

k.      Speech Language

l.       Voice

m.    Aerodigestive disorders

n.      Other

13.  It has become difficult to perform basic science research

a.      Not applicable

b.      Strongly disagree

c.      Disagree

d.      Neutral

e.      Agree

f.       Strongly agree

14.  It has become difficult to perform basic science research

a.      Not applicable

b.      Strongly disagree

c.      Disagree

d.      Neutral

e.      Agree

f.       Strongly agree

15.  It has become difficult to perform clinical research

a.      Not applicable

b.      Strongly disagree

c.      Disagree

d.      Neutral Agree

e.      Strongly agree

16.  Funding for research has become difficult

a.      Not applicable

b.      Strongly disagree

c.      Disagree

d.      Neutral Agree

e.      Strongly agree

17.  My funding for research comes from (May check more than one)

a.      I have no funding

b.      Institutional grants

c.      Society grants

d.      Industry

e.      Divisional or departmental grants

f.       NIH

g.      Other:

18.  My institution has a board that oversees clinical research protocols to ensure the protection of human subjects (IRB)

a.      Yes

b.      No

19.  My salary is dependent on (check any that apply)

a.      number of publications

b.      external funding

c.      number of abstracts

d.      number of presentations

e.      none of the above

f.       Other:

20.  My division provides the following support (check any that apply)

a.      research coordinator

b.      Someone to help me with IRB

c.      Someone to help me with stats

d.      Someone to help me with grant submissions

e.      Seed grants

f.       Protected time

g.      None of the above

h.      Other:

21.  What is the greatest barrier for your research career? (check all that apply?)

a.      Funding

b.      Protected time

c.      IRB

d.      Lack of collaborators

e.      Lack of a mentor

f.       Need for further training in grant writing

g.      Need for further training in study design and statistics

h.      There are no barriers

i.       Other:

22.  How Can ASPO help your research career? (Check all that apply)

a.      Provide grants for research

b.      Directory of mentors, contact info and their research interests

c.      Statistical and Study Design help

d.      Directory of Potential collaborators with their contact information and research interests

e.      Panel on how to develop a successful research career

f.       Workshops on grant writing or research success

g.      Network for multi-institutional collaboration

h.      I do not need any help

i.       Other:

23.  Do you have any suggestions or comments?

Appendix B: Survey distributed to ASPO members to determine barriers to a successful research career in 2021 (ASPO COVID Research Survey)

We are conducting a survey among ASPO members to learn about the effects of COVID-19 on research. The survey is twenty-two questions long and shouldn’t take longer than 10 minutes to fill out, and we will be very grateful for your response.

1.      How old are you? *Short answer text box*

2.      When did you complete your training? e.g. 2005   *Short answer text box*

3.      Gender

Female

Male

Prefer not to say

4.      Years in Practice?

0-5 years

6-10 years

11-15 years

More than 15 years

5.      Please describe yourself

Pediatric Otolaryngologist, Fellowship Trained

Pediatric Otolaryngologist, Non-Fellowship Trained

Pediatric Otolaryngology Fellow           

Otolaryngology Resident

Other..

6.      How would you describe your practice (check all that apply)?

Academic or University

Private Practice

Managed care

Hospital setting

Other..

7.      What percent of your time did you spend on research prior to the COVID-19 pandemic?

Not at all

1-10% of my time

11-20% of my time

21-50% of my time

51-75% of my time

More than 75% of my time

8.       What percent of your time would you like to spend on research?

None

1-10% of my time

11-20% of my time

21-50% of my time

51-75% of my time

More than 75% of my time

9.      How important is a research career to you personally?

Not Important

1

2

3

4

5

Very Important

10.    If you do not participate in any research activities, please stop here

11.    The focus of my research is on (check all that apply)

Retrospective review of components of clinical practice

Prospective analysis of components of clinical practice

Systematic review or meta-analysis of a clinical question

Clinical trials

Basic science

Other:

12.    My field of research interest is in (check all that apply)

Airway

Hearing Loss

Dysphagia

Otology

Professional Practice

Outcomes

Rhinology or Sinus

Tonsil or Adenoids

Head and Neck

Sleep

Speech/Language

Voice

Aerodigestive disorders

Other:

COVID-19-Specific Questions:

13.    Has the amount of time you spend on research changed since COVID-19?

Slight Decrease

Decrease

Unchanged

Slight Increase

Increase

14.     It has become difficult to perform basic science research since COVID.

Not applicable

Strongly disagree

Disagree

Neutral

Agree

Strongly agree

15.    It has become difficult to perform clinical research since COVID-19?

Not applicable

Strongly disagree

Disagree

Neutral

Agree

Strongly agree

16.    Has your funding for research changed since COVID-19?

Slight Decrease

Decrease

Unchanged

Slight Increase

Increase

17.    How did COVID-19 impact data collection or your ability to conduct research? (check all that apply)

            Not at all

            Inability to perform remote research

            Halted research involving laboratory animals or cell cultures

            Lost work due to closure of university or research laboratory

            Shortage of PPE or research supplies

            Inability to enroll new participants in studies

            Reduction in staff and collaborative research

            I have had more time to conduct research

            Other:

18.    Was your research focus changed from non-COVID-19 research to COVID-19 research?

Not at all

Increase in COVID-19 research, continued non-COVID-19 research efforts

            Increase in COVID-19 research, halted non-COVID-19 research completely        

19.     How did the added responsibility of being a caregiver for your loved ones, children, or friends due to COVID-19 impact time dedicated toward research efforts?

            No impact

            I was able to spend less time on research because of this impact

            I was able to spend more time on research because of this impact 

20.     Did COVID-19 change your research aims due to the fear of putting others and loved ones at an increased risk of transmission?

            Yes

            No

21.    The cancelation of conferences or lack of mentorship affected my ability to conduct research

Not applicable

Strongly disagree

Disagree

Neutral

Agree

Strongly agree

22.    Please add any comments you wish to share. ______________________

## References

[1] Sumi E, Murayama T, Yokode M (2009). A survey of attitudes toward clinical research among physicians at Kyoto University Hospital. BMC Med Educ.

[2] Askew DA, Clavarino AM, Glasziou PP, Del Mar CB (2002). General practice research: attitudes and involvement of Queensland general practitioners. Med J Aust.

[3] Ko CY, Whang EE, Longmire WP Jr, McFadden DW (2000). Improving the surgeon's participation in research: is it a problem of training or priority?. J Surg Res.

[4] Mansi A, Karam WN, Chaaban MR (2019). Attitudes of residents and program directors towards research in otolaryngology residency. Ann Otol Rhinol Laryngol.

[5] (2017). Barriers and attitudes to research among residents in plastic and reconstructive surgery: a national multicenter cross-sectional study. J Surg Educ.

[6] MacKinney EC, Chun RH, Cassidy LD, Link TR, Sulman CG, Kerschner JE (2015). Factors influencing successful peer-reviewed publication of original research presentations from the American Society of Pediatric Otolaryngology (ASPO). Int J Pediatr Otorhinolaryngol.

[7] Caudwell KM, Soranzo A, Lim LW, Aquili L (2022). How have COVID-19 stringency measures changed scholarly activity?. Ann N Y Acad Sci.

[8] Woolston C (2021). Job losses and falling salaries batter US academia. Nature.

[9] Rucker BM, Sajjadi NB, Brame LS, Vassar M, Hartwell ML (2022). The impact of COVID-19 on otolaryngology research: a cross-sectional analysis of discontinued trials. J Osteopath Med.

[10] Trecca E, Marano PG, Gelardi M (2021). Is 2020 the golden year of otolaryngology research? The impact of COVID-19 on the Italian academic production. Acta Biomed.

[11] Chillakuru YR, Gerhard EF, Shim T (2022). Impact of COVID-19 on otolaryngology literature. Laryngoscope.

[12] Schreiber MA, Differding J, Esposito TJ (2008). Research: questions and answers from academic trauma surgeons. J Trauma.

[13] Anker SD, Butler J, Khan MS (2020). Conducting clinical trials in heart failure during (and after) the COVID-19 pandemic: an Expert Consensus Position Paper from the Heart Failure Association (HFA) of the European Society of Cardiology (ESC). Eur Heart J.

[14] Allevi F, Dionisio A, Baciliero U (2020). Impact of COVID-19 epidemic on maxillofacial surgery in Italy. Br J Oral Maxillofac Surg.

[15] Jain A, Jain P, Aggarwal S (2020). SARS-CoV-2 impact on elective orthopaedic surgery: implications for post-pandemic recovery. J Bone Joint Surg Am.

[16] (2020). Elective surgery cancellations due to the COVID-19 pandemic: global predictive modelling to inform surgical recovery plans. Br J Surg.

[17] Liu N, Chee ML, Niu C (2020). Coronavirus disease 2019 (COVID-19): an evidence map of medical literature. BMC Med Res Methodol.

[18] Okafor S, Tibbetts K, Shah G, Tillman B, Agan A, Halderman AA (2020). Is the gender gap closing in otolaryngology subspecialties? An analysis of research productivity. Laryngoscope.

[19] Parker K: Modern Parenthood (2024). Modern Parenthood. Center’s Soc. Demogr. Trends Proj.

[20] Livingston G (2024). For most highly educated women, motherhood doesn’t start until the 30s. https://www.pewresearch.org/short-reads/2015/01/15/for-most-highly-educated-women-motherhood-doesnt-start-until-the-30s/.

[21] Krukowski RA, Jagsi R, Cardel MI (2021). Academic productivity differences by gender and child age in science, technology, engineering, mathematics, and medicine faculty during the COVID-19 pandemic. J Womens Health (Larchmt).

[22] Myers KR, Tham WY, Yin Y (2020). Unequal effects of the COVID-19 pandemic on scientists. Nat Hum Behav.

[23] Deryugina T, Shurchkov O, Stearns J (2021). COVID-19 disruptions disproportionately affect female academics. AEA Pap Proc.

[24] Gardner AK, Sudan R, Sidhu R, Mann BD, Scott DJ (2015). The association for surgical education CESERT grant program: the first 15 years. Am J Surg.

[25] Olaleye DO, Odaibo GN, Carney P (2014). Enhancement of health research capacity in Nigeria through north-south and in-country partnerships. Acad Med.

[26] Katz JS, Martin BR (1997). What is research collaboration?. Res Policy.

[27] Atieno AV, Onyancha OB, Kwanya T (2022). Trends, patterns and determinants of research productivity at the Technical University of Kenya. Inf Dev.

[28] Donnelly DT, Nicksic PJ, Zeng W, Dingle AM, Poore SO (2023). Evaluation of a full-time microsurgeon educator on resident training, research collaboration, and grant funding. J Reconstr Microsurg.

[29] Heaton-Shrestha C, Ooms A, Brady M, Pedley G, Bacon I, Strong S, Dundas J (2023). Interventions to enhance the research productivity of academic staff in higher education schools of nursing: A systematic review. Nurse Educ Pract.

